# Diversity in TAF Proteomics: Consequences for Cellular Differentiation and Migration

**DOI:** 10.3390/ijms150916680

**Published:** 2014-09-19

**Authors:** Jekaterina Kazantseva, Kaia Palm

**Affiliations:** 1 Protobios LLC, Mäealuse 4, Tallinn 12618, Estonia; 2 Cellin Technology LLC, Mäealuse 4, Tallinn 12618, Estonia; 3 The Department of Gene Technology, Tallinn University of Technology, Akadeemia tee 15, Tallinn 12618, Estonia

**Keywords:** TAF4, cell-specific transcription, alternative splicing, cancer stem cells

## Abstract

Development is a highly controlled process of cell proliferation and differentiation driven by mechanisms of dynamic gene regulation. Specific DNA binding factors for establishing cell- and tissue-specific transcriptional programs have been characterised in different cell and animal models. However, much less is known about the role of “core transcription machinery” during cell differentiation, given that general transcription factors and their spatiotemporally patterned activity govern different aspects of cell function. In this review, we focus on the role of TATA-box associated factor 4 (TAF4) and its functional isoforms generated by alternative splicing in controlling lineage-specific differentiation of normal mesenchymal stem cells and cancer stem cells. In the light of our recent findings, induction, control and maintenance of cell differentiation status implies diversification of the transcription initiation apparatus orchestrated by alternative splicing.

## 1. Introduction

Cell-specific differentiation for tissue reconstruction is an extensively studied area of research. Common mechanisms of body development by consequent induction of appropriate paracrine and transcription factors are well characterized. Although tissue- and cell-specific transcription factors are widely studied, the role of general transcription machinery in tissue development and homeostasis is not well understood. Next to the spatiotemporal activity of general transcription factors that governs cell differentiation processes, another core process with significant influence on cell-fate decision is alternative splicing. Besides generating protein diversity, coordinated regulation of a myriad of developmental mechanisms in the body is accomplished through the diverse array of alternative protein isoforms and non-coding RNAs. Herein, we focus on reviewing the role and functional changes in the core transcription factor complex IID (TFIID) in controlling pluripotency and lineage-specific differentiation of normal and cancer stem cells. Being at the top of a hierarchical structure that governs vital cell functions, core transcription complex subunits, including TAF4, present a valuable target in understanding cell differentiation and tissue formation, and for advancing tissue-engineering approaches of the clinic.

## 2. The Diversity of Transcription Factor Complex IID (TFIID) Complex Subunits in Organism Development

**Figure 1:**
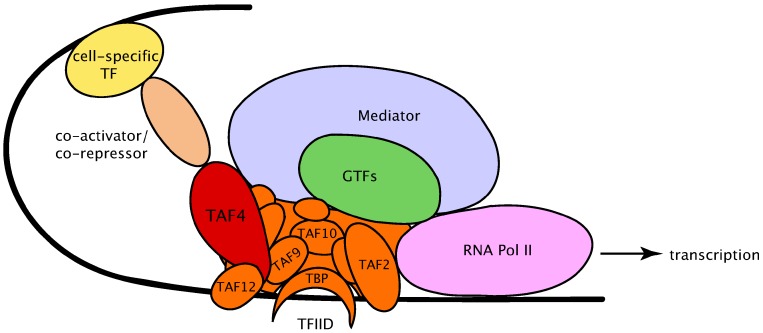
Multi-subunit tissue-specific RNA Pol II preinitiation complex (PIC) at the site of transcription. The main components of basal transcription preinitiation machinery (RNA Pol II (purple), GTFs (green), and Mediator (blue)) are presented. The tentative placement of some TBP-associated factors (TAF subunits) in the canonical TFIID complex (orange) is shown. TAF4 is specifically highlighted in brown. The co-activators/co-repressors (light brown) bridge upstream DNA-bound cell-specific transcription factors (yellow) with transcription machinery. It should be noted that various PIC complexes with different subunit compositions and sequence variation (isoforms) might be present, thus further diversifying PIC architecture beyond the example shown in this figure. TF, transcription factor; TBP, TATA box-binding protein; GTFs, general transcription factors.

Initiation of transcription is the first critical step in the regulation of gene expression. It requires simultaneous operation of a large cohort of transcriptional players—co-activators, trans-activators and components of the preinitiation complex (PIC), participating in the core promoter recognition [1,2] (Figure 1). However, the general transcription machinery that consists of RNA Pol II and the general transcription factors (GTFs) TFIIA, TFIIB, TFIID, TFIIE, TFIIF and TFIIH is necessary and sufficient for basal (core-promoter mediated) transcription.

TFIID is one of the several general transcription factors that compose PIC. TFIID is a large multi-subunit complex that together with TBP participates in the core promoter recognition [3]. TBP-associated factors (TAFs) were biochemically identified as stably associated with TBP components and originally named according to their electrophoretic mobility values [4]. Besides TBP, TFIID contains up to 14 different TAF subunits. The core composition of TFIID is well conserved from yeast to mammals. Interestingly, in addition to TBP, at least two TAFs bind to core promoters in a sequence-dependent manner [5]. Other components of the TFIID complex recognise multiple regulatory *cis*-elements in various combinations, as well as interact with modified nucleosomes and have enzymatic activities [6,7].

Different genes utilize unique composition and structural architecture of TBP and TAF subunits, suggesting mechanisms of cell-type-specific regulation of basal transcription factors [8]. The universality of TBP was questioned when TBP-related factors, including TRF1, TRF2 and TRF3, were discovered [9]. TRF1, which is highly homologous to TBP, represents a subunit of an alternative core promoter complex that in *Drosophila* directs promoter-selective transcription via different polymerases in a subset of tissues, including nervous system and gonads [10,11]. TRF2, although broadly conserved from *Caenorhabditis elegans* to human, does not bind to TATA box sequences and is unable to recruit canonical TFIID complexes [12,13]. TRF2 is required for early embryonic development [14] and is highly expressed in mouse testis [15]. These observations imply that only a limited set of core promoter recognition complex subunits, such as TBP and TRF2, is needed to produce highly varied expression patterns. Although TRF3 (TBP2) is widely expressed in adult mammalian tissues, its role is crucial for oogenesis and in early embryonic development [16]. Developmental studies of *Xenopus* have demonstrated that TRF3 can partially rescue loss of TBP, suggesting different activation mechanisms rather than recruitment of specific transcription complexes [17]. TRF3 is essential for the initiation of hematopoiesis [18] and together with TAF3 is required for differentiation of myotubes [19].

Additionally to core TAFs, TAF paralogs are expressed in different cells and tissues [20]. For example, TAF4b [21] together with TAF7L [22] were initially discovered as B-cell-specific, and are involved in oogenesis and spermatogenesis and share similarities in domain structure with respective TAFs. Association of TAF7L and TBP governs adipogenesis through the binding with PPARG-RXR cofactors and directing adipocyte-specific differentiation [23]. Five homologues of canonical TFIID subunits are expressed during spermatogenesis in *Drosophila*. Namely, the Cannonball, homologue of TAF5; No hitter, homologue of TAF4; Meiosis I arrest, homologue of TAF6; Spermatocyte arrest, homologue of TAF8; and Ryan express, homologue of TAF12 [24]. As a rule, up-regulation of expression of the paralog leads to the dynamic down-regulation of its core partner with accompanying changes in TAF sub-complex structure and composition.

Despite emerging data on the diverse composition of general transcription complexes and their heterogeneity of components during development, very little is known of the orchestration of the basal transcriptional activity in tissue-specific differentiation.

## 3. The Dynamics of TFIID Complex Components in Development and Differentiation

Given that the diversity of TFIID complexes in development is vast, their composition and functional dynamics at different stages of development are poorly understood.

At the earliest stage of vertebrate development, in the fertilised egg, two dynamic processes are highly crucial: zygotic gene activation and degradation of maternal mRNAs. Both of these events are regulated by the TBP activity [25]. Up-regulation of nuclear TBP levels promotes zygotic gene activation and the ratio of nuclear to cytoplasmic TBP protein regulates gradual degradation of cytoplasmic maternal mRNAs. Basal transcription complex dynamics in zygotic gene activation of *C. elegans* has been characterised for TAF5, TAF10 and TAF11 subunits [26]. In two-cell stage, TAF5 was identified to be vital for the open-complex formation, whereas TAF10 and TAF11 were detected in four-cell-stage nuclei with no impact on RNA Pol II activation. Similarly, in zebrafish embryos, both TBP and TAFs are highly expressed during early phases of gastrulation and their levels drop sharply at later stages of development [27].

Interaction of the intact basal transcription machinery with a set of tissue-specific transcription factors was previously suggested to be required for embryonic development and differentiation. However, more recent publications argue that lineage-specific differentiation involves the selective loss of some of the common RNA Pol II core complex subunits [9]. In addition, expression of different TFIID subunits is comparatively lower in non-differentiated cells as compared to fast proliferating cells [20]. Whether TFIID subunits are actively degraded or replaced by paralogs is currently not clear. Individual TAFs interact with upstream transcription factors and require specific core complex-specific partners for their functional activity. Consistent with this, canonical TFIID complexes do not contribute to transcription of the majority of genes in terminally differentiated hepatocytes [28]. TRF3–TAF3 complexes drive hematopoiesis and myogenic differentiation [18,29], and are essential for endodermal lineage commitment [30]; TAF4 controls ATRA-dependent differentiation [31], and TAF8 is involved in adipogenesis [32]. On the other hand, different transcription initiation mechanisms co-exist in cells during differentiation. Expression of some genes requires most of the canonical TFIID complex subunits, while others are transcribed by subsets of TFIID components. For example, expression of TAF8 is not detected in preadipocytes but is up-regulated during adipogenic differentiation, when the expression of other TAFs is down-regulated [32]. TAF10 is required for normal liver development [33]. Reduced expression of Taf1 and Taf4b affects proliferation of mouse embryonic maxillary mesenchymal cells and causes aberrant bone formation [34].

Germ cells are the best-characterised model of the transcription machinery adaptation during differentiation [35,36]. So far, the most diverse set of TBP and TAF paralogs is found in germ cells, supporting the concept of specialised TFIID complexes that are distinct from the canonical forms. For example TAF4b, the first identified tissue-specific TAF, is important for ovarian follicle development and function [37]. Male germ cell-specific TAF7L governs male fertility [38]. Expression of meiotic genes in gametes is controlled by TRF2 [39]. Oocyte-specific TRF3 replaces core TBP in these highly specialised cells, and its expression decreases during fertilisation, being substituted by TBP [40].

On the whole, very different models of transcription initiation operate during embryonic development and in the adult organism, supporting the concept of different transcription regulatory networks in proliferating and differentiating cells [41]. Identity of ESCs is governed by a set of sequence-specific pluripotent transcription factors, including OCT4, MYC, KLF4 and NANOG [42]. These factors control the activity of the basal transcription complexes via specific interactions with co-activators. Herein, TFIID activity contributes the most to the induction and maintenance of the highly plastic pluripotent state of ESCs [43]. Recent studies in mouse ESCs revealed preferential binding of TFIID to nucleosomes with the active epigenetic H3K4me3 and H3K14ac marks [44] and at genomic regions spanning transcription start sites of mouse ESCs [45]. Similar to early stages of development, in ESCs and pluripotent stem cells, active canonical TFIID complexes are required for cell function. The majority of TAFs are expressed in ESCs at high levels. TAF3 and TAF4 are specifically required for maintaining their pluripotency and function [30,46]. In contrast, different TFIID sub-complexes with redundant components are present in terminally differentiated cells, thereby reflecting the need of different cell types to respond to different external signals. High plasticity and adaptability of the core complex factors allow both highly specialised and broad initiation of transcription depending on the cellular context and developmental setup.

## 4. TATA Box-Binding Protein (TBP)-Associated Factor (TAF4): Structure, Function and Regulation

TAF4 is one of the largest and ubiquitously expressed subunits of the TFIID complex and is important for maintaining stability and integrity of the TFIID complex. Historically, TAF4 was the first TATA-binding associated factor with demonstrated co-activator function. Now, it is documented that TAF4 interacts with many activators, including Sp1 [47], CREB [48], NCoR [49], E-box transcription factors [50], and c-Jun [51]. TAF4 transcriptional activation is potentiated by the AF-2 motif of RARα, vitamin D3 and thyroid hormone receptors [52]. TAF4 interacts with HP1α and HP1γ, but not HP1β, further complicating its regulatory mechanisms and functions [53].

**Figure 2:**
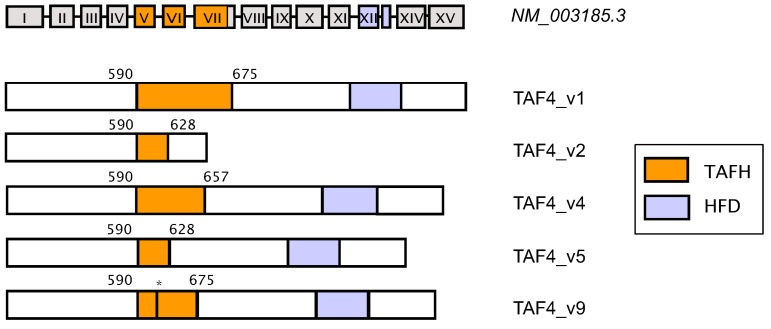
Alternative splicing of human *TAF4* (*NM_003185.3*) leads to a variety of protein isoforms. *TAF4* gene structure and major TAF4 isoforms with deletions in the co-activator-binding hTAF4–TAFH domain are outlined. Some isoforms of TAF4, for example TAF4 ASV_v2, differ in their *C*-terminus. Functionally important TAFH (hTAF4–TAFH, orange) and HF (Histone-fold, blue) domains are highlighted. Boundaries of hTAF4–TAFH domain are shown by amino acid residue numbers above the structures and correspond to their position in the canonical form of TAF4, where the * symbol represents deletion of the amino acids 628–658 in the TAFH domain.

The molecular structure of TAF4 is remarkably conserved throughout evolution. Human TAF4 protein consists of an *N*-terminal metazoan-specific polyglutamine tract region, central co-activator binding TAFH domain (ETO, CRI), and *C*-terminal histone-fold domain (HFD, CRII) (Figure 2) [54]. TAF4 HFD is conserved from yeast to human and plays a critical structural role in the TFIID complex formation. By analogy with histone H2A and H2B, TAF4 interacts with TAF12 through structurally similar HFDs. Formed octamer-like sub-structures support the integration of TAF4 into TFIID and its binding to the core promoter regions in the chromatin. The length of the DNA sequence, which is recognised by the TAF4/TAF12 dimers, is about 70 bp and is half of the length of nucleosomal DNA. This suggests the formation of hetero-complex structures, similar to the nucleosome complex assembly [55]. TAF4 HFDs are sufficient to nucleate the assembly of holo-TFIID complexes. In metazoans, TAF4–TAFH domain is highly conserved in all TAF4 and ETO family members [54]. Structural studies revealed that the α-helical folds of TAF4–TAFH domain forming a large hydrophobic groove are responsible for protein-protein interactions. Using the phage display screening method, the amino acid sequence that is targeting TAF4–TAFH binding surface was determined as DΨΨζζΨΦ (where Ψ represents V, I, L, or M; ζ represents hydrophilic residues including N, Q, S, or T; and Φ represents V, I, L, F, W, Y, or M) and was found to be present in different transcriptional regulators [54]. Thus, potential TAF4–TAFH binding partners include PBX proteins, important in limb development and hematopoiesis; HCF-1 dependent ZF and LZIP activators participating in chromatin modification and cell proliferation; Mediator subunit MED23 involved in post-translational remodeling of chromatin substrates; and a number of histone deacetylases, demethylases and kinases regulating cell cycle. Different to ETO–TAFH, vertebrate TAF4–TAFH domains exhibit unique packing of 5 helices and as a result, present very flat and extended binding surfaces. This allows a much broader spectrum of interactions and thus serves as a platform for positive and negative transcription regulation. It is important to note here that the *TAF4* gene is duplicated in mammals and has an ovarian-specific paralogue *TAF4b* [37].

The role of TAF4 in a range of cellular physiological processes and in somatic cell reprogramming has been extensively studied and identified. In various organisms, downregulation of TAF4 activity has dramatic effects on cell fate. RNAi-mediated knockout of *Taf4* in *Drosophila* considerably disturbs TFIID stability, suggesting that TAF4 is its key structural subunit [56]. In *C. elegans*, depletion of TAF4 is highly destructive with effects similar to RNA Pol II loss-of-function [57]. Reversible and controlled repression of TAF4 is one of the mechanisms that regulate cellular development and differentiation. In the early embryo of *C. elegans*, TAF4 interacts with OMA-1/2, proteins that are necessary for oocyte maturation. This interferes with TAF4 and TAF12 dimer formation and ultimately results in TAF4 sequestration to the cytoplasm. During the early stages of germline blastomere development, this process represses transcription. However, the degradation of OMA proteins in cytoplasm releases TAF4, which binds TAF12 and the resultant TAF4/TAF12 heterodimers translocate to the nucleus to restore transcription [57]. In mouse, *Taf4* is not essential for cell viability, but its inactivation affects proliferation [58]. Notably, *Taf4*^−/−^ fibroblasts contain intact TFIID and do not exhibit cell cycle arrest or apoptosis. Interestingly, *Taf4* inactivation is associated with alterations in cellular morphology, serum-independent autocrine growth and deregulation of more than 1000 genes [58]. These changes in gene expression together with suppression of serum-independent growth are restored by re-expression of protein isoforms containing the CRII domain. *Taf4* depletion leads to high expression of TGFβ thereby enhancing SMAD (Sma and Mad Related Family) signalling by the positive feedback loop. Loss of *Taf4* further induces the expression of matrix metalloproteases, CTGF and OPN, which are important regulators of metastasis, thereby contributing to the oncogenic functions of TAF4. Ectopic expression of truncated forms of TAF4, containing TAFH but not CRII domains, yielded in accelerated cell growth. In contrast, cells expressing TAF4 CRII alone display slowed rate of growth [59]. The levels of TAF4 expression have been shown to control cell differentiation. Targeted proteolysis of Taf4 is observed in differentiating mouse F9 embryonal carcinoma cells [60,61], and during differentiation of C2C12 myoblasts into myotubes [62,63]. In contrast, enhanced expression of Taf4 impairs endodermal differentiation, whereas enhanced expression of CRII domains had no effects on this differentiation process in F9 cells [31]. These results showed for the first time that regulated degradation of TAF4 is required for differentiation into select cell lineages.

TAF4 activity is vital for different cellular physiological processes. TAF4 was demonstrated to act as a co-factor of retinoic acid receptors, and that its CRII domain alone is sufficient to mediate CREB and RAR activity. In a case of CRII deletion, it has been shown that ATRA can signal via TP53 to exert anti-apoptotic effects [31]. In addition, TAFH was identified as a domain targeted by Pygopus that promotes WG/WNT target gene transcription throughout *Drosophila* development [64,65]. Selective loss of *Taf4* in the mouse fetal epidermis results in aberrant skin appearance and histology, enhanced water loss and early post-natal death, suggesting defective skin barrier function [59]. In adult mouse epidermis, Taf4 participates in the normal hair cycle, as Taf4 deficiency results in fur loss. In addition, Taf4 deficit induces epidermal hyperplasia and aberrant differentiation of mouse adult basal keratinocytes. Moreover, Taf4 inactivation significantly alters cell adhesion, cell communication and induces the expression of markers correlating with oncogenic transformation. These changes stimulate tumour formation by enhancing malignant transformation. Upon histological analysis, the tumour cells exhibit melanocyte-like phenotype with high expression of genes involved in melanocyte signalling. However, it is worth noting that a set of genes affected by the loss of *Taf4* in mouse keratinocytes is different from that seen in the embryonic fibroblasts or fetal epidermis, suggesting involvement of different regulatory pathways. For example, in fibroblasts, the TGFβ pathway is activated, whereas in keratinocytes the EGF signalling is enhanced. Other studies connect *Taf4* inactivation in MEFs with the formation of fibrospheres and activated expression of pro-oncogenic Collagen 6A3 [66]. The mechanism involves repression of Hippo signaling and activation of the WNT pathway. Interestingly, treatment of MEFs by ATRA restores TAF4-abolished effects.

The most recent studies have focused on the function of TFIID, and TAF4 in particular, in reprogramming of somatic cells and ESCs. ChIP sequencing data revealed the binding of Nanog and Oct4 to the regulatory region of mouse *Taf4* gene [67], which acted as an ESC-specific enhancer. In MEFs and adult human fibroblasts, exclusion of TAF4 from the TFIID complex inhibited reprogramming of the somatic cells while TAF4 over-expression facilitated iPSC formation [46]. These findings evidence a positive feedback circuit between TAF4 and the pluripotency factors. Namely, enforced expression of TAF4 activates expression of the pluripotency factors, which in turn enable to maintain high levels of TAF4 expression.

Thus implicated in the majority of vital cellular processes, TAF4 as the component of the general transcriptional machinery is a valuable target for controlling cell functions.

## 5. Coordinated Switching of *TAF4* Alternative Splice Variants Controls Differentiation and Migration of Normal Progenitors and Cancer Stem Cells

Alternative splicing plays a key role in generating complex proteomes, breaking the “one gene, one protein” rule. Existence of multiple mRNA variants for a single gene explains, at least in part, the complexity of some organisms like humans, having about 20,000 protein-coding genes in their genome [68]. Moreover, alternative splicing enables quantitative gene control through regulation of various regulatory RNAs and by targeting RNAs to non-sense-mediated decay. Genome-wide analysis has revealed differential expression of alternative spliced mRNAs in various tissues and cell types [69,70].

Consistent with the notion that alternative splicing should avoid destruction of the protein domains, it targets mostly the areas of structure with minimally exposed hydrophobic surfaces and with high intrinsic disorder [71,72]. Removal of protein–protein interaction domains by alternative splicing affects drastically protein function. However, changed by alternative splicing protein–protein interaction domains are very common to regulators of transcription [73,74]. Ankyrin repeat, DNA-binding zinc finger, homeobox, and KRAB [75] domains of transcriptional factors are frequent targets of alternative splicing modification.

To date, a close link between transcription and splicing has been shown [76]. Transcription factors such as TAT-SF1, CA150, SKIP and co-activator PGC-1 are present in the spliceosome and perform dual functions in transcription and splicing. Thus, it is highly conceivable that both processes are simultaneously coupled together in space and time [77]. Consistent with this, the *C*-terminal domain of RNA Pol II directly participates in exon recognition [78]; differences in promoter structure recognition are often associated with differences in alternative splicing of pre-mRNA [79]; some transcriptional co-activators and co-regulators modulate alternative splicing, sometimes in a synergetic manner [80,81].

One of the major challenges today is studying the role of specific splice variants in the cell context. It has been shown that siRNA targeting of intron or exon sequences near the alternative-splicing sites affects the splicing process of these sequences [82]. Among protein isoforms whose function is studied by using this technique are adapter protein ShcA [83], spleen tyrosine kinase [84], and pyruvate kinase M1 and M2 [85].

Analyses of the alternative splicing of *TAF4* in various mouse and human cells and tissues identified multiple and complex splicing patterns [86,87]. In mouse, five alternative isoforms with deletions in the functional domains result in dominant negative effects in nuclear receptor-mediated transcriptional activation [86]. Complex patterns of *TAF4* alternative splicing occur in different human tissues [87]. Although, a significant number of *TAF4* alternatively spliced mRNAs contain a premature termination codon, indicating that these splice transcripts are subjected to the nonsense-mediated RNA decay, others preserve the open reading frame (ORF) and could be considered as possessing some functional properties in the cell. Depending on the cell type, there is a different balance of expression between full-length *TAF4* and the rest of the alternative splice variants (ASVs). Some of the ASVs are invariantly expressed across tissues; others exhibit patterns of cell type-specific expression. Interestingly, the dominant expression of canonical full-length form of TAF4 is observed mainly in the cells with stem cells characteristics [86,87,88], while differentiated cells demonstrate multiple patterns of different combinations of *TAF4* ASVs. The most prone to alternative splicing exons of *TAF4* are those encoding the functional co-activator-binding hTAF4–TAFH domain (Figure 2). Since this domain is involved in the cooperation with a set of transcription activators like E-box proteins, WNT signalling mediating Pygopus, and others, it is likely that the structure of hTAF4–TAFH, which is cell-specifically targeted by alternative splicing, affects its interaction properties and dictates further cell development. As mentioned above, TAF4 is one of the structural components of TFIID complex and affects its stability. Therefore, alternative splicing of *TAF4* is most likely to have strong impacts on the integrity and functional activity of TFIID as well as on the core transcription machinery as a whole. Consistent with this, some of its alternative transcripts generate alternative protein isoforms, which differ from canonical TAF4 protein functions.

Our recent data demonstrate that *TAF4* RNAi leads to the appearance of new ASVs with serious effects on cell differentiation [87,88]. Namely, in human adipose-derived mesenchymal stem cells, silencing of canonical TAF4 activity and appearance of new hTAF4–TAFH-defected ASVs contributes to the repression of adipogenic and ostegenic programs, while promoting chondrogenesis [87]. Similarly, high expression of full-length *TAF4* is necessary for maintenance of multipotency in normal human neural progenitors but is dispensable for transcription in differentiated cells, as *TAF4* RNAi induces expression of ASVs and stimulates neuronal differentiation [88]. The exact mechanisms are currently unclear, although, our initial findings revealed the involvement of WNT and p53 signalling pathways. Our data showed the switching from canonical to non-canonical WNT pathways in response to *TAF4* RNAi [87]. It is interesting to note that other studies have found that suppression of canonical WNT signalling supports chondrogenesis, contributes to cell migration [89,90], and affects bone formation and limb development [91]. Thus, the switch from canonical to non-canonical WNT signalling could be one of the regulative mechanisms in TAF4 activity-deficient progenitor cells. p53 signalling appears also to play a role in differentiation of TAF4-deficient mesenchymal stem cells. Several recent studies link TP53 activity to cell differentiation [92] in addition to its well-established role in apoptosis. Interestingly, in TAF4-depleted mesenchymal stem cells (MSCs), increased levels of TP53 induce cell cycle arrest without any signs of apoptosis or cell senescence [87].

Adding support to the hypothesis that sustained expression of TAF4 impairs cell differentiation, *TAF4* RNAi induced spontaneous differentiation of facial dermal fibroblasts into melanocyte-like cells [93]. Phenotypic features of melanocyte-specific transformation of TAF4-deficient fibroblasts included darkening of the cell pellets due to melanin secretion and high levels of expression of master of melanogenic transcription factor MITF. This molecular transformation was accompanied by events reminiscent of the epithelial-to-mesenchymal transition, as *TAF4* silencing inversely correlated with cadherin switch: down-regulation of TAF4 in the dermal fibroblasts suppressed *E*-cadherin and supported *N*-cadherin expression. It is known from other data that cadherin switch contributes to cancer progression and emergence of cells with stem-like characteristics [94,95].

Cells that are prone to multilineage differentiation, such as fibroblasts and mesenchymal stem cells, express canonical TAF4 at low levels as compared with wide expression of alternative splice variants encoding TAF4 isoforms. At the same time, cells with high pluripotent potential express elevated levels of canonical TAF4. In contrast, cancer cells, such as melanoma, express both canonical and other isoforms of TAF4 at high levels, thereby raising the question of the differentiation potential of tumour cells. Overall, highly plastic and heterogeneous melanoma cells fit well to the cancer stem cell model. Melanoma cells express pluripotent and differentiation-associated genes and differentiate into a wide range of cell types [96,97,98]. Altogether, our data using *TAF4* gain- and loss-of function studies allow the conclusion that melanomas have properties of stem cells and exhibit multilineage differentiation potential [93]. Through regulation of the *TAF4* ASVs expression balance in melanoma, it is possible to get melanoma populations with strong multipotent properties or drive cells to differentiate. Upon *TAF4* RNAi, a change in expression from canonical to alternative TAF4 isoforms with disturbed hTAF4–TAFH activity is observed, whereas removal of hTAF4–TAFH activity accelerates differentiation of melanoma cells along chondrogenic, adipogenic and neural lineages. The accelerated differentiation is accompanied with the down-regulated expression of melanoma-specific genes and cellular proliferation. In contrast, ectopic expression of the canonical TAF4 in melanoma cells leads to the up-regulation of pluripotency markers KLF4, OCT4 and NANOG [93]. Thus, pluripotency and differentiation status of normal and cancer cells can be controlled by *TAF4* ASV expression.

Targeting the self-renewal and differentiation potential of stem cells for clinical use is worthless if the migration of cells to target tissues cannot be appropriately controlled. Migration of stem cells to different organs and target niches requires active guiding, a process termed homing. Homing is necessary for tissue transplantation and for seeding stem progenitors during development. Being better understood and studied for hematopoietic stem cells, it is also applicable to mesenchymal and cancer stem cells [99,100]. Furthermore, considering that cancer is often a stem cell-retaining disease, it is important to understand the different features of homing and migration of cancer and normal stem cells in order to better control tumour progression. However, the molecular mechanisms underlying the motility potential of stem/progenitor cells are not well studied.

**Figure 3:**
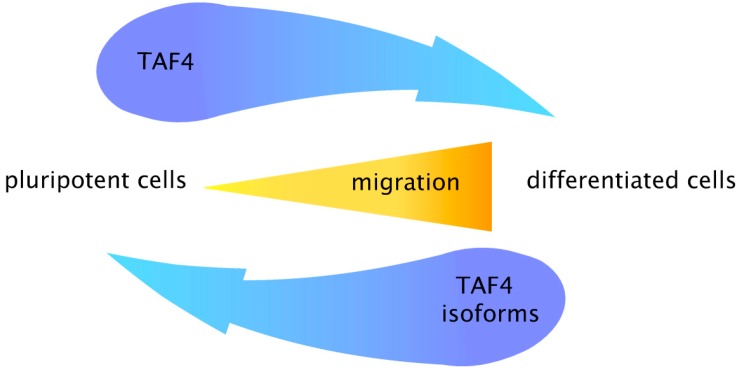
A model describing the cross-talk between canonical TAF4 and its alternative isoforms in pluripotent (stem cells) and differentiated cells, and their relation to migration. High levels of canonical TAF4 (blue arrow from left to right) are a characteristic to normal stem cells and cancer cells. In contrast, differentiated cells maintain high expression of TAF4 isoforms with deletions in hTAF4–TAFH domain (blue arrow from right to left). Functional balance between canonical and alternative isoforms of TAF4 is crucial for cell migration. Consistent with this, cells with low expression of canonical TAF4 are prone to migration contrasting with low motility of cells with high levels of TAF4 activity.

Our recent data demonstrate that reduced expression of full-length *TAF4* by RNAi leads to the enhanced motility of normal dermal fibroblasts and malignant melanoma cells [93]. In contrast, transient over-expression of canonical TAF4 diminishes the invasion potential of the melanoma cells. Observed changes were accompanied by molecular switches in E/N cadherin and matrix metallopeptidases expression. The regulated expression of canonical and other TAF4 isoforms reveals a remarkable conservation in different cell systems, and supports cell state transitions (between pluripotency and differentiation) and migration (Figure 3).

Altogether, our studies identify TAF4 as a critical modulator of cell differentiation and metastatic spread of cancer. Loss of hTAF4–TAFH activity by regulated alternative splicing affects cell homeostasis of normal and cancer cells. Regulation of the equilibrium of *TAF4* alternative mRNAs encoding functionally different protein isoforms provides the possibility to direct cell status towards either pluripotency or differentiation. This finding is reinforced by the observation that motility of normal and malignant cells and the expression of full-length *TAF4* are inversely correlated, thereby combining the concepts of cell migration in development and disease progression.

## 6. Conclusions

Deeper understanding of the molecular mechanisms behind cellular differentiation is extremely important for the development of new and improved cellular therapies. One of the novel approaches could include modulation of the composition and activity of basal transcription complex factors, for example, utilizing *Taf4* RNAi to enhance chondrogenic differentiation. This strategy has two significant benefits: suppression of TAF4 canonical activity accelerates chondrogenic differentiation and enhances motility of the mesenchymal progenitors, thereby directing modified cell pools to the distant target sites throughout the body.

In summary, the detailed understanding of TAF4 proteomics in different cellular contexts, including tumour cells, will clearly be of immense benefit for future prospects of cell-directed therapeutics.

## Abbreviations

ATRA: all-trans retinoic acid
BAF45/BAF53: BRG1-Associated Factor 45/53
CA150: co-activator of 150 kDa
ch-ERG: chicken *Ets*-related transcription factor
CREB: cAMP response element-binding protein
CTGF: connective tissue growth factor
c-Jun: V-jun avian sarcoma virus 17 oncogene homolog
EGF: epidermal growth factor
ETO domain: 8;21 translocation domain
GTFs: general transcription factors
HCF-1: host cell factor 1
HP: heterochromatin protein
KLF4: Krüppel-like factor 4
KRAB: Krüppel associated box
lncRNA: long non-coding RNA
LZIP: leucine zipper protein
MEFs: mouse embryonic fibroblasts
MIA/CD-RAP: melanoma inhibiting activity/cartilage-derived retinoic acid-sensitive protein
MITF: microphthalmia-associated transcription factor
MYC: myelocytomatosis viral oncogene
NCoR: nuclear receptor co-repressor
OCT4: octamer-binding transcription factor 4
OMA-1/2: overlapping activity with M-AAA protease 1/2
OPN: osteopontin
PBAF: polybromo-associated BAF
PBX: pre-B cell leukemia transcription factor
PGC-1: peroxisome proliferator-activated receptor-gamma coactivator
PPARG: peroxisome proliferator-activated receptors
RARα: retinoic acid receptor-alpha
RXR: retinoid X receptor
ShcA: Src homology 2 domain containing transforming protein A
SKIP: Ski-interacting protein
SMAD: Sma and Mad Related Family
Sox9: SRY (sex determining region Y)-box 9
Sp1: specificity protein 1
SWI/SNF: SWItch/Sucrose NonFermentable
TAF: TATA box-binding protein (TBP)-associated factor
TAT-SF1: cofactor required for Tat activation of HIV-1 transcription
TBP: TATA-box protein
TGFβ: transforming growth factor beta
WG/WNT: wingless
ZF: zinc finger
